# Practical Notes and Formulæ

**Published:** 1878-03-20

**Authors:** 


### *PRACTICAL NOTES AND FORMULÆ.*

### PNEUMONIA ABORTED BY VERATRUM VIRIDE.

Dr. A. G. Hobbs writes: I do not claim anything original in the treatment of pneumonia with veratrum viride, but I do claim what I think only the minorty of the profession now believes—that veratrum viride, if begun in time, and persisted in sufficiently, will abort many cases of pneumonia. The damp, cool weather of tne fall and winter just passed has been prolific in the production of pneumonia; indeed, it has been almost the sole disease that I have had to combat during the last few months, having at times as many as seven and eight cases on hand. But my object is to report nine cases, that I have called aborted cases of pneumonia, and to recall the attention of some of the readers of your journal to the value of veratrum viride in the treatment of pneumonia.

'Tis an old song, I will admit, but one that I consider worthy of repetition. In all the nine cases, I began the treatment immediately, which I regard as a *sine qua non* in the use of this drug.

Nearly all of these cases began with a "bad cold," and a few hours previous to my first visit, a rigor. Upon examination, I found the quick pulse, the high temperature, the short breathing, the flushed cheeks, the dry cough, the pain in the side, and, upon ausculation, the subcreptant and sometimes the creptant rale.

After administering a cathartic, I begin immediately with a solution of quinine and Norwood's tincture, and repeat it in large doses every hour, till the pulse is lowered, or till emesis ensues. When the former, I desist till the pulse begins to rise again; when the latter, I give a small dose of laudanum in ice water to allay the stomach, and after a few hours, if the pulse remains high, return to the solution, and give as before.

After eighteen to twenty-four hours the pulse becomes nearly natural, the temperature is lowered, and the flush has left the cheek. If the fever returns, the solution is again resorted to, and, in most cases, for the last time it is needed. All the smyptoms subside except the cough. An expectorant is given to assist in throwing off the sputa, which is but slightly tinged, and the patient is convalescent in from 36 to 48 hours of alternate treatment and rest.

I do not say that all cases of pneumonia will succumb to this treatment. I have found it so in nine out of twelve cases seen immediately after the first symptoms have set in. 'Tis true that all these cases were in children, and it is further true that pneumonia attacks children much more lightly than adults.

In the three remaining cases, the disease went through all its stages in spite of the treatment, and finally succumbed to quinine and carb. ammon.

I was, at first, almost incredulous myself at such results; and in order to confirm my diagnosis, I tested the sputa and urine taken simultaneously, and found that the former contained chlo. sodium, and the latter none, which differentiated the diagnosis between pneumonia and bronchitis.

If my observations have been true and my conclusions correct, these nine cases never reached the second stage; hence, they can be properly called "aborted."

### CINCHONIA.

Dr. J. Maul writes: I have been recently testing samples of the above article, prepared by Messrs. Powers & Weightman, Philadelphia.

In a case of menorrhagia, of long and obstinate character, which had resisted all the usual agents, the patient being much reduced, feeble and anæmic, I prescribed as a tonic the cinchonia, combined with sugar of milk and bicarb. of soda, as in the sample furnished me, and was most agreeably disappointed and surprised to find my patient in a few days greatly relieved, the remedy having proved very efficacious, not only by its tonic property, but by equalizing the circulation, also checking the menorrhagia.

Several weeks have elapsed, and there is no return of the disease. The dose given was four to five grains every three hours during the day, acidulous drinks being allowed. I will mention that the remedy in this case seemed to have a decided anodyne property.

In another case of malarial fever, attended with a dumb chill every day, I prescribed successfully—

Cinchonia, grs. iv.

every two hours, giving three doses previous to the chill; relieved in four days.

I am testing the remedy in a third case, a low grade of typho-malarial fever, attended with slight remissions every morning. In two days use of the remedy, I notice a perceptible improvement, there being a decided prolongation of the morning remission. I have again noticed the anodyne effect above mentioned.

[Information received since the above note was sent us, reports that the last case mentioned yielded to the remedy in a few days.—Ed.)

### CONSTIPATION.

A writer in the *Clinic* recommends cascara sagrado as an admirable remedy.

R.—Fluid ext· cascara sagrado............oz. ss.
Aq. cinnamon......................oz iss.
Sac. alb..............................dr ii.

M. Teaspoonful before each meal.

Another:

R.—Sodæ sulphatis......................20 grs.
Ac. nitro muriatic.................5 drops.

Sig.—Take half hour before breakfast in half glass of water. —*Brief.*

### POST PARTEM HEMORRAHGE.

In a case of secondary post partem hemorrhage, nine days after delivery, we introduced two fingers, removed the clots, and the os being sufficiently dilated, we inserted therein a pledget of cotton, saturated with tincture of iodine, and over this a tampon, and applied a compress and bandage firmly upon the abdomen, and gave ergot internally. In 48 hours the tampon and pledget were removed, and no further trouble occurred.—Ed.

### EVAPORATING LOTION.

As an evaporating lotion for an inflamed breast, or for hyperæmia of the conjunctiva where there is heat and swelling of the eyelids, the following is very useful:

R.—Spiritus etheris nitrici,.................dr i.
Acidi acetici aromat....................gtt vi.
Aquæ distil..........................oz vi.

M. To be sponged over the part three or four times per day.

### HYDROCELE.

Dr. Lubin, in a British journal, suggests the following as an injection after operation for hydrocele, as giving rise to much less pain than the old method of using the iodine alone:

R.—Tinct. iodine com...................oz. iij.

Chloroformi ...........................dr ijss.

M.

### EXCELLENT EYE WATER.

R.—Zinci sulphatis......................gr iv.
Morphiæ sulphatis..................gr ij.
Atropiæ sulphatis...................gr ss.
Aquæ rosæ..........................oz j

Use as a collyrium.

Dr. R. E. Hutchins writes: If the following will be of any benefit to the numerous readers of your valuable journal, I herewith submit it to you for publication:

For worms in a child two or three years old:

R.—Santonin,
Hyd. chlor. mit.................aa grs xij.

M. and divide into six powders. Sig.—Give one powder morning, noon and night for two days, and on the morning following last dose, give a full dose of castor oil.

I will state that I have used the above prescription for worms nearly two years, and have never known it to fail in a single instance.

Bolton, Miss., February, 1878.

